# Supplementary material for: Using the R Package Spatstat to Assess Inhibitory Effects of Microregional Hypoxia on the Infiltration of Cancers of the Head and Neck Region by Cytotoxic T Lymphocytes
Source: Cancers (Basel). 2021 Apr 16;13(8):1924. doi: 10.3390/cancers13081924 (PMC8072547; doi:10.3390/cancers13081924)
Supplement: Supplementary file 1 [file cancers-13-01924-s001.zip › cancers-1152627-Suppl-Table-1.pdf]

Supplementary Tables

Supplementary Table 1. Antibodies (AB), IHC protocol and resulting staining patterns.

Antigen	Primary AB (Cat.-No., clone, species, dilution, incubation)	Primary AB supplier	Fluorochrome (Cat.-No.)	Fluorochrome supplier	Staining pattern
**CA IX**	ab108351, EPR4151[2], rabbit: 1 : 200, for 1 h at 28 - 30°C	Abcam, Cambridge (UK)	FITC (46410)	Thermo Fisher Scientific, Waltham, MA, USA	Membranous
**CD8**	M7103, CD8/144B, mouse (mono), 1:50, for 1 h at 28 - 30°C	DAKO, Denmark A/S (DK)	TSA Plus Cyanine 3 (NEL744001KT)	PerkinElmer, Waltham, MA, USA	Membranous
**CD73**	13160/D7F9A, rabbit, 1:100, for 1 h at 28 - 30°C	Cell Signaling Technology	TSA Plus Cyanine 5 (NEL745E001KT)	PerkinElmer, Waltham, MA, USA	Membranous

Supplementary Table 1: Details of the staining protocol regarding the primary antibodies, catalogue number, species, dilution and incubation time, as well as the fluorochrome coupled with the primary antibody and the expected staining patterns.
