# Supplementary figures and images for: Using the R Package Spatstat to Assess Inhibitory Effects of Microregional Hypoxia on the Infiltration of Cancers of the Head and Neck Region by Cytotoxic T Lymphocytes
Source: Cancers (Basel). 2021 Apr 16;13(8):1924. doi: 10.3390/cancers13081924 (PMC8072547; doi:10.3390/cancers13081924)

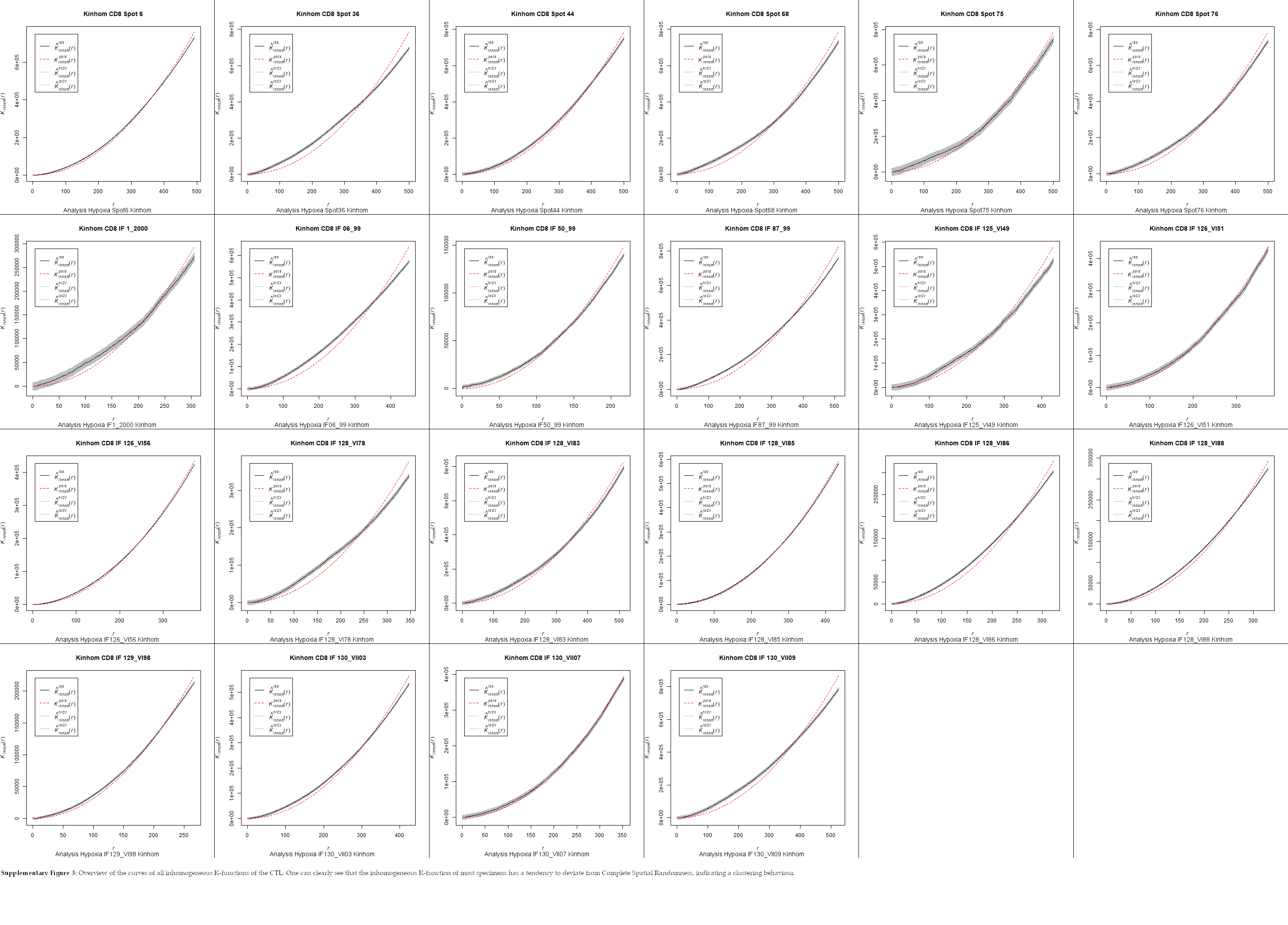

Supplement: Supplementary file 1 [file cancers-13-01924-s001.zip › cancers-1152627-Suppl-Fig-3.tif]

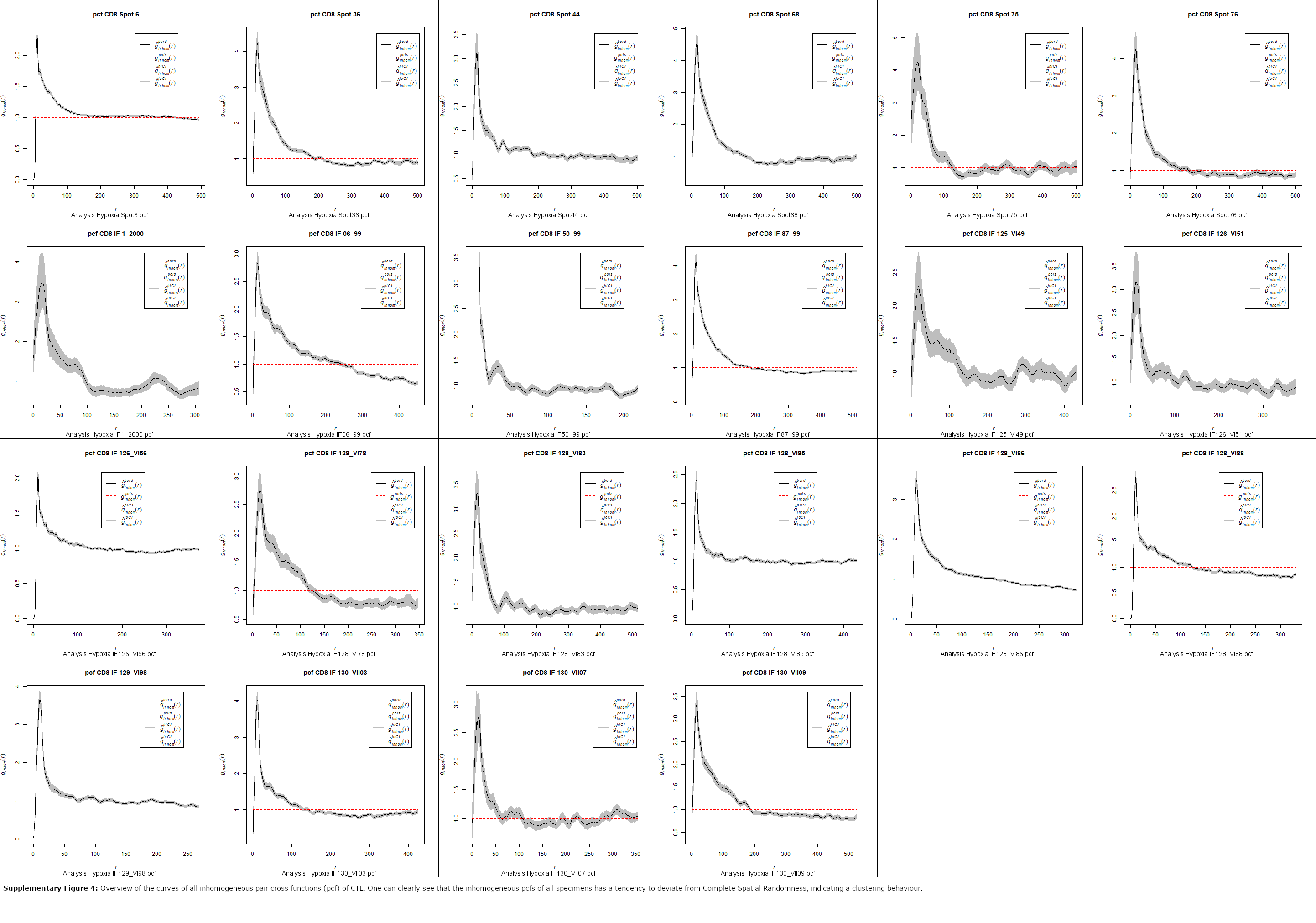

Supplement: Supplementary file 1 [file cancers-13-01924-s001.zip › cancers-1152627-Suppl-Fig-4.tif]

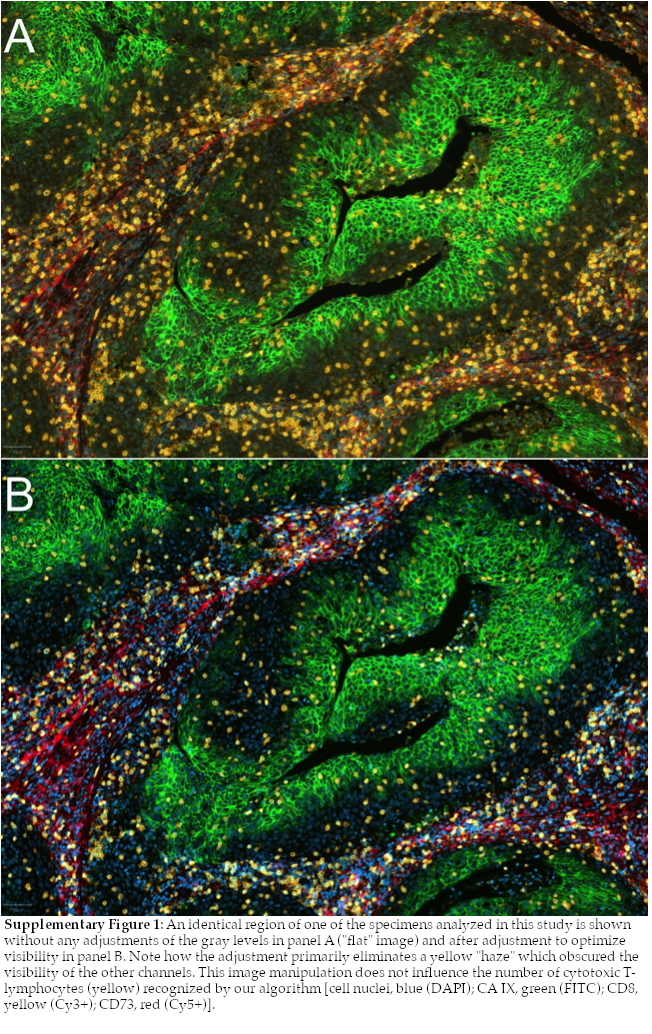

Supplement: Supplementary file 1 [file cancers-13-01924-s001.zip › cancers-1152627-Suppl-Fig-1.png]

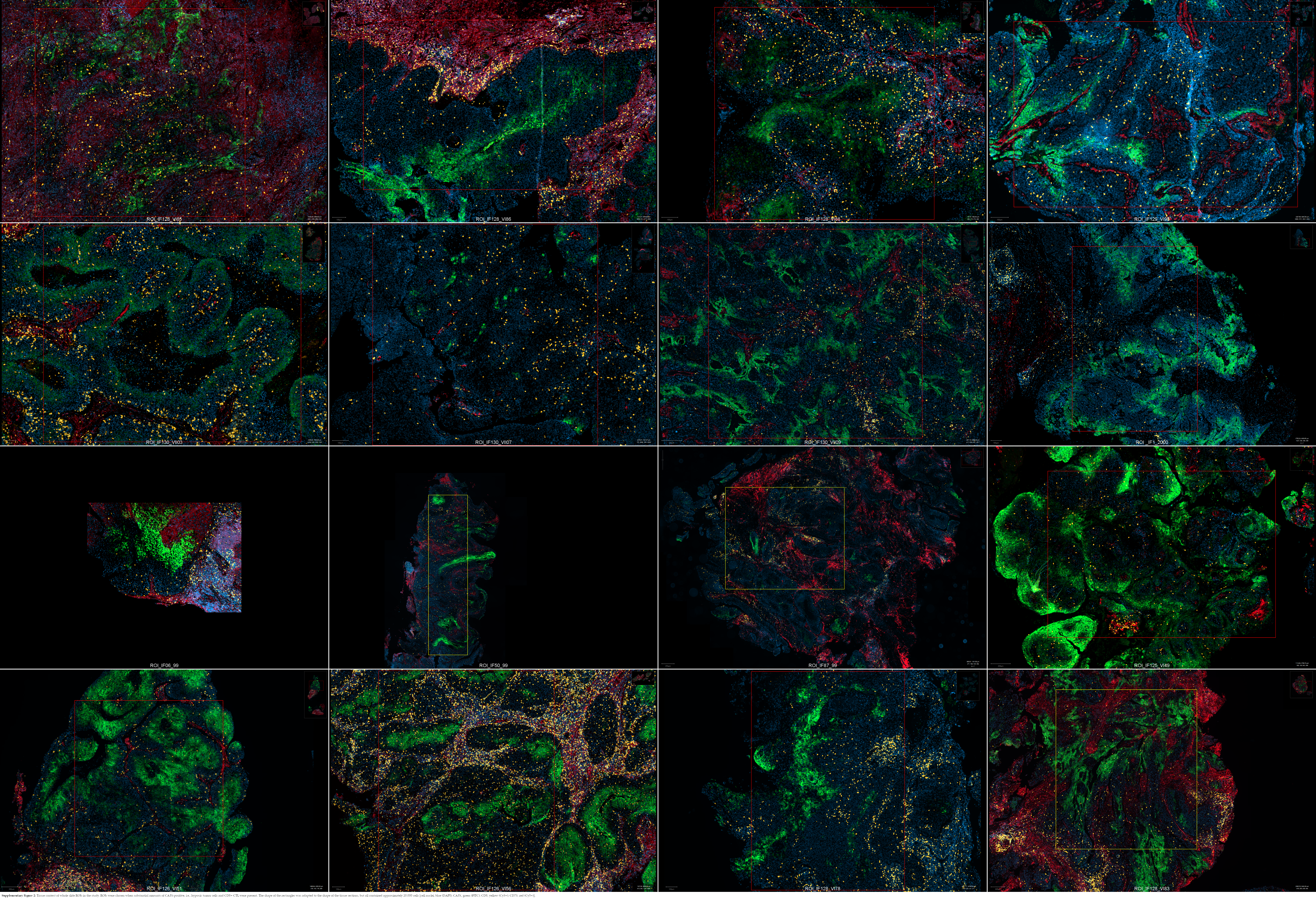

Supplement: Supplementary file 1 [file cancers-13-01924-s001.zip › cancers-1152627-Suppl-Fig-2.png]
